# Sequential Pulsed Light and Ultrasound Treatments for the Inactivation of *Saccharomyces cerevisiae* and PPO and the Retention of Bioactive Compounds in Sweet Lime Juice

**DOI:** 10.3390/foods13131996

**Published:** 2024-06-25

**Authors:** Lubna Shaik, Snehasis Chakraborty

**Affiliations:** 1 Department of Food Engineering and Technology, Institute of Chemical Technology, Matunga, Mumbai 400019, India; lubna.shaik@dypiu.ac.in; 2 School of Biosciences and Bioengineering, D Y Patil International University, Akurdi, Pune 411044, India

**Keywords:** flavor compounds, microbial structure, vitamin C, *Saccharomyces cerevisiae*, polyphenol oxidase

## Abstract

Designing a pasteurization con dition for sweet lime juice while ensuring microbial safety, enzymatic stability, and high nutritional quality is crucial for satisfying stakeholder demands. The present research investigates the effects of matrix pH, ultrasound treatments, and sequential pulsed light on the microbial population, enzyme activity, and bioactive chemicals in sweet lime juice. The sequential pulsed light (PL: 0.6–0.84 J/cm^2^) and ultrasound (US: 0.2–0.4 W/cm^3^) treatments for sweet lime juice were optimized using response surface methodology (RSM). A three-factor full factorial design was used for this purpose. The independent variables encompassed pH (X_1_), PL effective fluence (X_2_, J/cm^2^), and US intensity (X_3_, W/cm^3^). The responses assessed included the inactivation of *Saccharomyces cerevisiae* (Y_1_, log cfu/mL) and polyphenol oxidase (PPO: Y_2_ in %) and the retention of vitamin C (Y_3_, %). The polynomial models were optimized using numerical optimization to attain the maximum desirability value (0.89). The optimized PL + US sample (0.8 J/cm^2^ + 0.4 W/cm^3^, respectively) at pH 3.5 resulted in a 5-log cycle reduction in *S. cerevisiae* count and a 90% inactivation in PPO activity and retained 95% of its vitamin C content. This optimized sample underwent further analysis, including phenolic profiling, assessment of microbial cell morphology, and examination of enzyme conformational changes. After sequential pulsed-light (0.8 J/cm^2^) and ultrasound (0.4 W/cm^3^) treatments, yeast cells showed unusual structural changes, indicating additional targets besides membranes. Following PL + US treatment, the PPO composition changed to 2.7 ± 0.1% α-helix, 33.9 ± 0.3% β-sheet, 1.4 ± 0.2% β-turn, and 62 ± 0.7% random coil. Impressively, the optimized PL + US sample maintained a sensory acceptance level similar to that of the untreated sample.

## 1. Introduction

Sweet lime (*Citrus limetta*), also called mosambi, is rich in antioxidants, phenolics, and vitamin C. The consumption of sweet lime juice is renowned for its potential to treat scurvy, diabetes, dehydration, and skin diseases [1]. It is preferred for its mild flavor and unique aroma. However, this juice is susceptible to both microbial and enzymatic spoilages, negatively affecting its quality and limiting its shelf life. Pectin methylesterase (PME) is the enzyme responsible for cloud loss in sweet lime juice. To ensure microbial safety in fruit juice, the U.S. Food and Drug Administration recommends a 5-log_10_ cycle reduction in the pathogen population [2]. Furthermore, the extent of microbial inactivation in a matrix is regulated by its pH [3]. Depending on maturity, sweet lime juice has a pH range from 3.5 to 4.5, and total soluble solids (TSS) vary between 7 and 12 °Brix [4]. The batch thermal pasteurization condition for the sweet lime juice is reported for 10 min at 80 °C [5]. Unfortunately, this severe thermal treatment condition also degrades heat-sensitive vitamins and other components in the juice, thus reducing its freshness. To address these needs, researchers across the world have started exploring the application of nonthermal processing technologies, for example, ultrasonication, plasma, pulsed electric field (PEF), high-intensity pulsed light (HIPL), high-pressure processing (HPP), and membrane filtration (MF). Recent studies revealed that nonthermal processing technologies, such as pulsed light (PL) and ultrasound (US) treatments, can preserve bioactive components in fruit juice while attaining food safety.

Pulsed light (PL) represents a nonthermal technology featuring white light generated by an inert gas lamp, ideally xenon. This light spectrum typically spans from 200 to 1100 nm in wavelength [6]. It can inactivate bacteria and enzymes while retaining nutrients [7]. PL is an excellent alternative for surface disinfection. In addition, the restricted light penetration in opaque juices, such as sweet lime juice, restricts the trials of scaling them up [7]. Likewise, ultrasound (US) is another nonthermal technique utilizing sound waves above the audible 20 kHz range. The US is generated through the conversion of electrical pulses into sound energy by transducers with the required frequency and intensity. As these US waves traverse through a medium, they create compressions and rarefactions, leading to a substantial increase in energy and mass transmission rate. Ultrasound’s cavitation impacts bacteria and enzymes in fruit juice [8]. Future research is required to develop automated, cost-effective, and energy-efficient commercial ultrasound systems while ensuring the maximum production of high-value and microbiologically safe juice. Microbiological safety, enzyme stability, and nutrient retention in PL or US-treated strawberry, orange, pear, and apple juices were already examined [9,10,11].

Several studies suggest that pH impacts microbial or enzyme inactivation during nonthermal treatments. There is not very much information available on thermal and nonthermal treatments of sweet lime juice. Pellicer et al. [12] explored the influence of matrix pH on the inactivation of PPO under pulsed light exposure. The authors concluded that the extent of PPO inactivation was faster at a low pH, and it has a significant impact on the unfolding of enzyme conformation. Namala et al. [13] examined the UV and thermal processing of sweet lime juice. Khandpur et al. [5] examined the degradation of nutrients in sweet lime juice after ultrasonication. Shaik and Chakraborty [14,15] explored how matrix pH affected the microbiological, enzyme, and qualitative features of PL- and US-treated sweet lime juice individually.

It is evidenced that various hurdle technologies work in synergy to inactivate microorganisms [16]. Ramírez-Corona et al. [17] conducted a study to determine the effect of US, UVC, and combined treatment targeting the inactivation of mold, yeast, and total aerobic mesophilic bacteria (TAMB) in samples of orange juice and mango nectar. It was concluded that the combined US + UVC treatment improved the log microbial reductions as compared to the samples that were individually treated with US or UVC. Sequential nonthermal processing inactivates bacteria and enzymes, but limits nutrition loss in the food. There is no research exploring how sequential nonthermal processing and matrix pH affect sweet lime juice-quality attributes. To explore it, a well-spaced experimental design and modeling are needed to optimize and statistically validate answers and understand how independent process characteristics (factors) affect them. Response surface methodology (RSM) is a common and reliable method for systematic experimental design and optimization. Using a simple quantitative optimization procedure, RSM helps us understand how factors affect responses and provides quick optimum outcomes [18]. This study examines how sequential pulsed light, ultrasound treatments, and matrix pH affect sweet lime juice’s microbial population, enzyme activity, and bioactive compounds. The conditions were fine-tuned through a combination of RSM and numerical optimization techniques. The hypothesis is that combining two or more hurdles (such as PL and US along with a reduction in matrix pH) can produce microbially safe and enzymatically stable juice at lower intensities of either treatment. Moreover, the attributes of equivalent thermally pasteurized sweet lime juice were analyzed for comparison. Quantifying the influence of matrix pH on sequential PL and US treatments’ lethality can help the juice processing industry identify the optimum pH–optimal fruit harvesting conditions.

## 2. Materials and Methods

### 2.1. Materials and Chemicals

Fresh sweet limes (*Citrus limetta*) were purchased from Matunga in Mumbai, India. The sweet lime variety with a TSS of 11.7–12.2 °Brix was considered. The microorganism strains were provided by NCL in Pune, India. Media agars were purchased from HiMedia Labs in Mumbai, India. Furthermore, chemicals such as anhydrous sodium carbonate, gallic acid, methanol, and 2,2-diphenylpicrylhydrazyl were provided by HiMedia Labs and Research-Lab Fine Chem Companies in Mumbai, India. The enzyme tyrosinase (PPO) [T3824-25KU] was purchased from Sigma-Aldrich in Bangalore, India.

### 2.2. Extraction of Sweet Lime Juice

**Figure 1:**
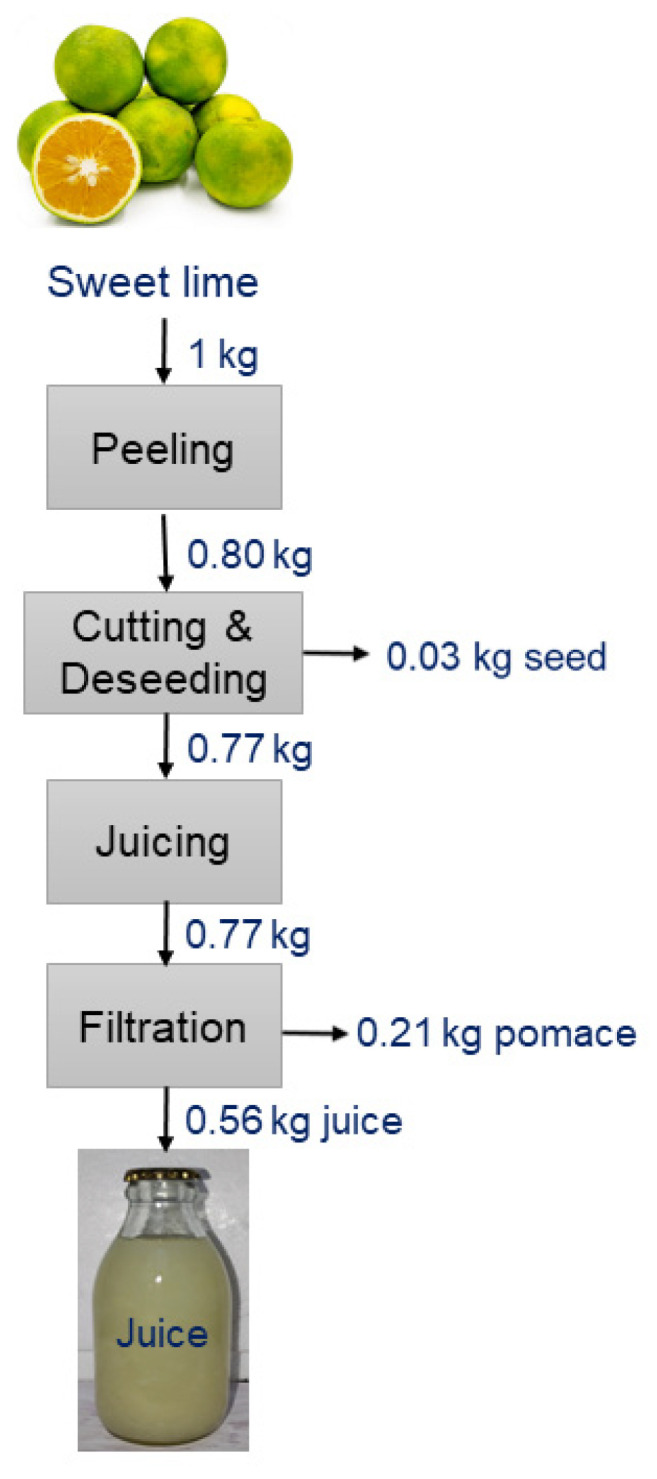
A detailed flowsheet for the extraction of sweet lime juice.

After being peeled, sweet limes were processed into juice using a centrifugal juicer (HR 1863/20 Philips, Chennai, India) at a speed of 5360× *g* and subsequently filtered through a 100-micron mesh. A detailed extraction flowsheet for sweet lime juice is presented in Figure 1. Citric acid and sodium bicarbonate solutions were employed to adjust the pH of the juice to 3.5, 4.0, and 4.5.

### 2.3. Experimental Design

The optimization of the sequential PL and US process was achieved through RSM. The experimental design utilized a three-factor–three-level (3^3^) full factorial design, with the three independent variables being pH (*X*_1_), PL effective fluence (*X*_2_, J/cm^2^), and US intensity (*X*_3_, W/cm^3^); the responses analyzed were inactivation of *Saccharomyces cerevisiae* (*Y*_1_, log cfu/mL), inactivation of PPO (*Y*_2_, %), and retention of vitamin C (*Y*_3_, %). Shaik and Chakraborty [14] reported that *S. cerevisiae* was the most resistant microorganism, even more so than *Escherichia coli*, *Listeria monocytogenes*, and natural microbiota, such as aerobic mesophiles and yeasts and mold. The trend was also true for the ultrasound treatment of the juice [15]. Both studies confirmed that, among the spoilage enzymes, polyphenol oxidase (PPO) was the most resistant to PL and US intensity, and vitamin C was the most sensitive bioactive component among other phytochemicals present in sweet lime juice. Therefore, regarding microbial safety, the survival count for *S. cerevisiae* was taken, and a >90% inactivation of PPO was desired to achieve the enzymatic stability of the juice. Moreover, ensuring a 5-log reduction in *S. cerevisiae* will also ensure a >5-log reduction in *E. coli*, *Listeria monocytogenes*, aerobic mesophiles, and yeasts and molds. The thermal pasteurization process, equivalent in efficacy, was conducted within a thermostatic water bath (AI-7981, I-therm, Mumbai, India) at a temperature of 95 °C for a duration of 5 min.

The independent variables (*X_i_*) were transformed into dimensionless coded values (*x_i_*) using Equation (1), where *X_max_* and *X_min_* denote the maximum and minimum values of *X_i_*, respectively.

(1) $$x_{i}=\frac{X_{i}-\frac{X_{max}+X_{min}}{2}}{X_{max}-\frac{X_{max}+X_{min}}{2}}$$

The boundary values of the experimental domain, which were determined through preliminary experiments and the referenced literature, were established. Specifically, the coded value domain ranged from −1 to +1 for pH (*X*_1_), PL effective fluence (*X*_2_), and US intensity (*X*_3_), with respective ranges of 3.5–4.5, 0.6–0.84 J/cm^2^, and 0.2–0.4 W/cm^3^. For each independent variable, three equidistant levels (*x_i_* = −1, 0, and 1) were considered, resulting in a total of 3^3^ experimental runs (including factorial points) to explore the entire domain. Additionally, five experiments were replicated at the center point of the domain (coded as 0, 0, 0) to assess the lack of fit. In addition, separate PL (0.6, 0.72, and 0.84 J/cm^2^ without any US treatment) and US treatments (0.2, 0.3, and 0.4 W/cm^3^ without any PL treatment) were performed at all three matrix pH levels (3.5, 4.0, and 4.5). 

### 2.4. Response Surface Methodology

A quadratic polynomial model (Equation (2)) was developed for each response, depicting its relationship with the independent variables in their coded forms.

(2) $$Y_{i}=\beta_{0}+\beta_{1}x_{1}+\beta_{2}x_{2}+\beta_{3}x_{3}+\beta_{4}x_{1}x_{2}+\beta_{5}x_{1}x_{3}+\beta_{6}x_{2}x_{3}+\beta_{7}x_{1}^{\;2}+\beta_{8}x_{2}^{\;2}+\beta_{9}x_{3}^{\;2}$$

In this context, *Y_i_* (where *i* = 1 or 3) represents the actual response value. The model’s regression coefficients are denoted as follows: *β*_0_ (constant term), *β*_1_, *β*_2,_ and *β*_3_ (coefficients for linear terms of x_1_, x_2_, and x_3_, respectively); *β*_4_, *β*_5_, and *β*_6_ (coefficients for interaction terms); and *β*_7_, *β*_8_, and *β*_9_ (coefficients for quadratic terms). Here, *x*_1_, *x*_2_, and *x*_3_ refer to the coded values of pH, PL fluence, and US intensity, respectively. The adequacy of the model fit was assessed using metrics such as the coefficient of determination (*R*^2^), adjusted *R*^2^, F-value, and *p*-value, along with evaluating the non-significant lack of fit (*p*_lof_). Additionally, response surface analysis was employed to visually represent the combined or interactive effects between any two parameters among pH, PL effective fluence, and US intensity on the experimental responses.

### 2.5. Numerical Optimization

For optimizing the processing conditions, the log cycle reduction in *S. cerevisiae* count, inactivation of PPO, and vitamin C retention were maximized. A minimum 5-log cycle reduction in the *S. cerevisiae* population and 90% inactivation in PPO activity was set as a target during optimization. The desirability of individual responses was correlated with the process parameters according to Equation (3).

(3) $$d_{i}=\frac{Y_{i}-L_{i}}{U_{i}-L_{i}}$$

In this context, *d_i_* represents the desirability index for *Y_i_*, where *L_i_* and *U_i_* are the lower and upper limits for *Y_i_*, respectively. To consolidate these desirability values, an overall desirability index (*D*) was computed using Equation (4).

(4) $$D={\left[\;{d_{1}}^{r_{1}}\times {d_{2}}^{r_{2}}\times {d_{3}}^{r_{3}}\right]}^{\frac{1}{r_{1}+r_{2}+r_{3}}}$$

Additionally, *r*_1,_ *r*_2_, and *r*_3_ denote the relative importance (rated on a scale of 1 to 5) of *Y*_1_, *Y*_2_, and *Y*_3_, respectively. A numerical optimization technique was employed to maximize the value of *D*, which falls within the range of 0 to 1 (with 1 being the most desirable) at any given combination of *X*_1_, *X*_2_, and *X*_3_ within the defined domain. The process conditions yielding the maximum *D*-value were considered the optimized conditions. Subsequently, these optimized conditions were validated through actual experimental trials.

### 2.6. Processing of Sweet Lime Juice

#### 2.6.1. Pulsed-Light Processing

Pulsed-light (PL) processing of sweet lime juice was conducted on a benchtop X-1100 System, according to Shaik and Chakraborty [14]. Throughout the PL treatment, a J-type thermocouple recorded the sample temperature (pencil type, length of 1 inch, diameter of 6.35 mm, Thermonic, Gujarat, India). The effective fluence of the PL treatment was maintained at 0.60, 0.72, and 0.84 J/cm^2^ by performing experiments at 2.8 kV/160 s, 2.9 kV/180 s, and 2.4 kV/240 s, respectively. The spectral distribution of flash lamps was 21% ultraviolet, 36% visible light, and 43% infrared. The pulse frequency was 1 Hz, and the ON/OFF duration (pulse width) was 400 μs. To measure the amount of fluence received by the sample, a pyroelectric energy sensor (PE-50C Ophir Optronics Solutions Ltd., Jerusalem, Israel) at the equivalent elevation was placed below the lamp house. The dosimetry for PL treatment was calculated as per the method suggested by Gómez-López and Bolton [19] (Table 1). The effective fluence (F_e_, J/cm^2^) per pulse is the fluence rate times pulse width. The fluence rate values for 0.60, 0.72, and 0.84 J/cm^2^ were 9.36 ± 0.05, 10.06 ± 0.02, and 8.74 ± 0.01 W/cm^2^, respectively (Table 1). When the PL treatment was conducted at 0.80 J/cm^2^, the condition was 2.7 kV/225 s with a fluence rate of 8.88 ± 0.02 W/cm^2^. Further on in this manuscript, the PL treatment has been recognized by its respective effective fluence (F_e_, J/cm^2^) value.

#### 2.6.2. Ultrasound Processing

A 250 W ultrasonic homogenizer (Model ATP-250, Probe sonicator, Athena Technologies, Mumbai, India) with a 9 mm probe was used for sonication. The interval between pulsed light and ultrasound treatments was one minute. Then, 50 mL juice samples were processed at 20 kHz. Extrinsic control parameters of US intensity at 0.2–0.4 W/cm^3^ (150–190 W) were varied by 6 s ON/3 s OFF pulses. The ultrasonic probe was placed 20 mm from the beaker’s bottom (4.8 cm beaker diameter and 3.4 cm height of the juice). The intensity for the US treatment was maintained at 0.2, 0.3, and 0.4 W/cm^3^ by performing experiments at radiofrequency power outputs of 150, 170, and 190 W, respectively.

#### 2.6.3. Selection of Sequence for Pulsed Light (PL) and Ultrasound (US) Treatments

For the selection of the sequence, the experiments were conducted at the four possible combinations of extreme levels of PL effective fluence and US power intensity (Table 2). Therefore, the experiments were PL (0.60 J/cm^2^) + US (0.2 W/cm^3^); PL (0.84 J/cm^2^) + US (0.4 W/cm^3^); US (0.2 W/cm^3^) + PL (0.60 J/cm^2^); and US (0.4 W/cm^3^) + PL (0.84 J/cm^2^) (Table 2). The pH of the juice was fixed at mid-pH 4.0. The same three responses, such as the survival count of *S. cerevisiae* (*Y*_1_, log cfu/mL), inactivation of PPO (*Y*_2_, %), and retention of vitamin C (*Y*_3_, %) in the juice, were determined after the sequential treatment.

### 2.7. Characterization of Optimized Sequential PL- and US-Treated Sweet Lime Juice

Various quality attributes of optimally sequential PL- and US-treated and thermally pasteurized juice samples were analyzed as discussed below. The attributes include microbial enumeration, determination of enzyme activity, and estimation of acidity, soluble solids, viscosity, color profile, total phenolics, antioxidant activity, and vitamin C content in the juice, together with its sensory acceptance. After the sequential PL + US treatment, the samples were cooled to 2 °C and analyzed for various attributes within 3 h.

#### 2.7.1. Microbial Enumeration

The maintenance of microbial stock cultures (*Escherichia coli* ATCC 43888, *Listeria monocytogenes* ATCC 13932, and *Saccharomyces cerevisiae* ATCC 9763), inoculum preparation, and inoculation in the juice were performed following the protocol described by Shaik and Chakraborty [14]. A total of 5 mL of the microbial cell suspension was inoculated in a 50 mL juice sample to attain the desired concentration of 7.0 ± 0.5 log cfu/mL. According to Guerrouj et al. [20], the detection of aerobic mesophiles (AM) and yeast and molds (YM) was carried out. In total, 10 cfu/mL of juice was chosen as the microbiological detection limit. 

#### 2.7.2. Enzyme Assay

Polyphenol oxidase (PPO) and peroxidase (POD) activity in sweet lime juice were determined following the method outlined by Shaik and Chakraborty [14]. Specifically, PPO activity was assessed at 420 nm, while POD activity was measured at 470 nm. Likewise, pectin methyl esterase (PME) was extracted, and its activity was quantified using 0.05% apple pectin as the substrate, following the procedure detailed by Sahoo and Chakraborty [21]. The protein concentration in the extract was determined using bovine serum albumin. PME activity, expressed as a single unit (U), represented the amount of crude extract required to produce one micromole of carboxylic group per minute per mL of the sample.

#### 2.7.3. Measurement of Physicochemical Properties

The total soluble solids (TSS) and titratable acidity (TA) of the juice samples were analyzed using a handheld refractometer (Erma Inc., Tokyo, Japan) and standard titration methods, respectively, according to Shaik and Chakraborty [14]. A pH meter was used to measure the pH of sweet lime juice. The Brookfield viscometer (AMETEK Brookfield India Centre of Excellence, Mumbai, Maharashtra, India) was used to measure the viscosity of the juice [22]. A sweet lime juice concentration of 100 mL was placed in a glass beaker, and the temperature was set at 25 °C. The torque was adjusted by selecting a specific spindle (RV-02) and its rotational speed of 60 rpm for a certain juice sample.

The color characteristics of the juice sample were analyzed using a Hunter-Lab colorimeter (LabScan-XE LX17375, Reston, VA, USA) in the CIE format, which represents the L*a*b* color space. The calculations of the total color change (ΔE*) and browning index (BI) involved the application of the following formulas, as per Shaik and Chakraborty [15]: Equation (5) for ΔE* and Equation (6) for BI.

(5) $$\Delta E^{*}=\sqrt{{\left(L_{t}^{*}-L_{u}^{*}\right)}^{2}+{\left(a_{t}^{*}-a_{u}^{*}\right)}^{2}+{\left(b_{t}^{*}-b_{u}^{*}\right)}^{2}}$$

(6) $$BI=\frac{31}{0.172}\;\left[\frac{a^{*}+1.75\;L^{*}}{5.645L^{*}+a^{*}-3.012\;b^{*}}\right]$$

In Equation (5), the ‘*t*’ and ‘*u*’ suffixes represent the respective indices for treated and untreated juice.

#### 2.7.4. Total Phenolic Content, Antioxidant Capacity, and Vitamin C

The assessments of vitamin C, total phenolic content (TPC), and antioxidant capacity (AOX) in the juice were carried out through spectrophotometric techniques, following the procedures detailed in the work of Shaik and Chakraborty [14]. Vitamin C content was expressed as grams of ascorbic acid (AA) per milliliter (mL) of juice, using L-AA as the reference compound. TPC was determined as grams of gallic acid equivalent (GAE) per mL of juice, with gallic acid serving as the reference compound. AOX, measured in grams of gallic acid equivalent antioxidant capacity (GAEAC) per mL of the sample, was quantified for each 1 mL of the sample. The percentage decrease in bioactive compounds in the treated sample was evaluated by comparing it to the untreated sample.

#### 2.7.5. Sensory Analysis

Sensory evaluation was conducted on juices that demonstrated enzymatic stability and microbial safety. The assessment involved a partially trained panel comprising 25 members, including 16 males and 9 females, aged between 23 and 35 years old, affiliated with the Institute of Chemical Technology in Mumbai. This panel evaluated the juices based on six sensory attributes: aroma, taste, color, consistency, mouthfeel, and aftertaste. The semi-trained panelists, who were not professional sensory experts, underwent a comprehensive 10 h training program spread over two weeks. This training covered various attributes and scales until their evaluations were consistently aligned. Each panelist assigned a hedonic score (*S*) to the juices on a scale ranging from 1 (dislike extremely) to 9 (like extremely). Additionally, they indicated the importance (*I*) of each sensory attribute, using a scale from 1 to 5, representing ‘not at all important’, ‘somewhat important’, ‘important’, ‘very important’, and ‘extremely important’, respectively. To determine the overall acceptability (*OA*) of each juice sample, Equation (7) was utilized, where ‘*n_a_*’ denotes the number of attributes, ‘*S*’ represents the hedonic score on the 1 to 9 scale, and ‘*I*’ indicates the corresponding importance score on the 1 to 5 scale.

(7) $$OA=\frac{1}{n_{a}}\times \frac{\sum \left(S\times I\right)}{\sum I}$$

*OA* represents the weighted average of the product of hedonic scores and importance. It nullifies the person’s individual bias to judge a sample based on a certain attribute. On the other hand, each sensory attribute cannot have the same importance during evaluation, as it varies from panelist to panelist. Therefore, *OA* combines all the scores for all six attributes and corresponding importance and provides a single value out of 9, making the comparison easier. 

#### 2.7.6. Phenolic Profile Using LC-DAD-ESI-MS/MS

The methodology developed by Rodriguez-Rivera et al. [23] was employed to extract phenolic compounds from sweet lime juice. After diluting the juice with an equal volume of 80% methanol (*v*/*v*), the resulting mixture was centrifuged at 7155× *g* for 5 min at a temperature of 6 °C. Prior to injection into the LC-MS/MS system (Agilent 1260 HPLC system; Agilent Technologies, Palo Alto, CA, USA), the extracts were filtered using a membrane filter with a pore size of 0.45 microns (manufactured by Whatman Inc., Clinton, NJ, USA). The HPLC equipment comprised a diode array detector, an autosampler (G1367 E, 1260 HIP ALS), a binary pump (G1312 B), a degasser (G1322), and another binary pump (G1312 B) (G1351D 1260 DAD VL). Phenomenex Luna reversed-phase C-18 column (4.6 mm 250 mm, 5 m) was employed (Torrance, CA, USA). Two solvents, solvent A, water/formic acid (99:1; *v*/*v*), and solvent B, acetonitrile/solvent A (60:40; *v*/*v*), made up the mobile phase. The procedure outlined by Kelebek et al. [24] was used to elute phenolic chemicals. In addition to exact reference compounds, the calibration of chemically similar substances was used for some instances while accounting for the molecular weight adjustment factor. 

#### 2.7.7. Morphology of *Saccharomyces cerevisiae*

To observe alterations in the morphology of *S. cerevisiae* cells following sequential treatment under optimized conditions, scanning electron microscopy (SEM) was employed. Initially, a culture of *S. cerevisiae* containing 7.1 log_10_ cfu/mL was mixed with 40 mL of citrate phosphate buffer at pH 4 and allowed to acclimate for 1 h. The control group consisted of untreated samples, while the treated *S. cerevisiae* cells in buffer at pH 4.0 were exposed to conditions including 0.80 J/cm^2^ (PL treatment only), 0.4 W/cm^3^ (US treatment only), and a combination of 0.80 J/cm^2^ and 0.4 W/cm^3^ (sequential PL + US treatment). Subsequent to the pulsed light treatment, the cells were recovered via centrifugation at 3913× *g* for 10 min at 20 °C. The retrieved cells were then subjected to fixation, dehydration, and freeze-fracturing following the protocol outlined by Kaláb et al. [25]. The fixation involved incubating the microbial cells with 2% glutaraldehyde for 18 h to serve as a fixative agent. Excess cells were rinsed with a 0.1 M sodium cacodylate solution for 5 min. Dehydration was carried out using a graded ethanol series (70%, 85%, and 100% (*v*/*v*)) to displace air spaces within the tissues. Lastly, liquid nitrogen was utilized for freeze-fracturing. The specimen was stored in a 50% (*v*/*v*) glycerol solution at 4 °C until SEM analysis was conducted. The imaging of freeze-fractured cells was performed using a low-vacuum scanning electron microscope (FEI™ Quanta 200, FEI Company, Hillsboro, OR, USA), with SEM images of both untreated and pulsed-light-treated *S. cerevisiae* cells acquired at 4000× and 2000× magnifications, respectively.

#### 2.7.8. Circular Dichroism Analysis of PPO

The PPO sample for circular dichroism (CD) analysis was prepared by dissolving it in a pH 6.5 SSP buffer solution, resulting in a final protein concentration of 1.98 µmol/L. Circular dichroism spectra were obtained using a JASCO J-720 CD spectropolarimeter (Japan Spectroscopic Company, Tokyo, Japan) equipped with a 1 mm optical path length quartz cuvette at a room temperature of 25 ± 1 °C. CD spectra were scanned in the far UV range (250–200 nm) with four replicates, utilizing a scanning rate of 50 nm/min and a 1 nm bandwidth. The CD data were expressed in terms of the mean residue ellipticity, [θ], in deg cm^2^ dmol^−1^, as computed using Equation (8).

[θ] = (0.1 × θ × MRW)/(E × d)(8)

where d represents the path length in cm, E denotes the PPO concentration (mg/mL), and θ signifies the ellipticity (mdeg). MRW stands for the mean residue weight, calculated as the protein’s mean weight (in atomic mass units or Da) divided by the number of residues in the protein. The molecular weight of PPO was considered as 119 kDa and the mean amino acid residue weight (MRW) for PPO was taken as 113.7 Da [26]. The secondary structure components were analyzed using DichroWeb: online analysis for protein circular dichroism spectra website (https://dichroweb.cryst.bbk.ac.uk/home.shtml (accessed on 25 April 2023)). The fundamentals and algorithm for DichroWeb analysis have been detailed by Miles et al. [27]. The K2D method, when combined with DichroWeb, was utilized to process circular dichroism spectra as the input and generate an estimation of the secondary structure composition (namely alpha helix and beta strand) for the corresponding protein [28]. K2D, a neural network, functions by linking neurons from an input layer to an output layer. The output layer, representing secondary structure, is derived from the input layer’s CD data through weighted connections to each neuron. During training, these weights are initially randomized. Both layers are supplied with extensive CD and structural data, similar to reference proteins. Through iterative adjustments, the weights are fine-tuned until an accurate secondary structure profile is achieved.

### 2.8. Statistical Analysis

Each run was conducted thrice, and each treatment was replicated three times, resulting in a total of nine data points for each treatment condition. Six data sets were collected for each PL + US condition for the microbial counts. The results are presented as mean values along with their respective standard deviations. To assess the significance of any changes in these mean values, a one-way analysis of variance (ANOVA) followed by Tukey’s HSD test with a 95% confidence interval was employed. Microsoft Office Excel (Microsoft Corporation, Redmond, WA, USA, 2016) was utilized for the purposes of response surface methodology (RSM) and numerical optimization.

## 3. Results and Discussions

### 3.1. Selection of Sequence for Pulsed Light (PL) and Ultrasound (US) Treatments

It is clear from Table 2 that the influence of the sequence of PL and US intensities has no statistically significant effect (*p* > 0.1) on its microbial and enzymatic lethality. For instance, after PL (0.84 J/cm^2^) + US (0.4 W/cm^3^), the log reduction in the *S. cerevisiae* population was 6.0, whereas a 5.9 log cycle reduction was obtained after a US (0.4 W/cm^3^) + PL (0.84 J/cm^2^) treatment. Therefore, for the optimization, juice samples were treated at the desired fluence level of PL followed by a specific US intensity. 

### 3.2. Effect of Sequential Pulsed Light (PL) and Ultrasonication (US) Treatments on S. cerevisiae, PPO, and Vitamin C

The intensity of sequential pulsed-light (PL) and ultrasonication (US) treatment had a significant influence on the inactivation of *S. cerevisiae* (*Y*_1_, log cfu/mL) and PPO (*Y*_2_, %). The initial population of *S. cerevisiae* in the juice was 7.1 log cfu/mL. The inactivation of *S. cerevisiae* ranged between 3.6 and 6.1 log cfu/mL when the sequential PL + US treatments were considered (Table 3). The PPO inactivation ranged between 36.1 and 100%. A microbially safe (>5 log cycle reduction in *S. cerevisiae* population) and enzymatically stable (>90% inactivation of PPO activity) juice was produced within the PL (0.60–0.84 J/cm^2^) + US (0.2–0.4 W/cm^3^) domain, where the vitamin C retention (*Y*_3_, %) varied in the range of 92–100% (Table 3). Overall, a higher microbial and enzyme inactivation was obtained at a lower pH when the PL and US intensities were the same. As expected, more PL exposure or an intense US treatment resulted in higher lethality toward *S. cerevisiae* and PPO in the juice of the same pH. An increase in voltages during PL treatment yielded increased fluence rates, which in turn impacted the total fluence since it is the fluence rate times the treatment time. In terms of response, only the total effective fluence has been considered. In individual PL and US conditions, the *S. cerevisiae* population decreased by a maximum of 5.5 and 2.9 log_10_ cycles at 0.84 J/cm^2^ and 0.4 W/cm^3^, respectively, when the juice pH was 3.5. In the case of sequential treatments, the corresponding yeast inactivation of 6.1 log_10_ cycles was achieved when juice samples were treated at 0.60 J/cm^2^ + 0.4 W/cm^3^ (pH 3.5). The sample temperature was 15 °C before the PL + US treatment, with a maximum temperature rise of 14.1 °C after the PL treatment and 11.1 °C after the US treatment, as summarized in Table 3.

#### 3.2.1. Response Surface Models

Response surface methodology (RSM) is employed to establish a connection between independent and response variables with the aim of pinpointing an optimal condition. In our experimental design matrix, the data were fitted to various polynomial models, and it was found that the quadratic polynomial (Equation (2)) provided the best fit, as evidenced by high *R*^2^ (>0.9), adjusted *R*^2^ values (>0.9), and an insignificant lack of fit (*p_lof_* > 0.1). Consequently, a quadratic polynomial model was developed to illustrate the impact of pH and PL + US process parameters on the dependent variables. Table 4 presents a summary of the regression coefficients and other parameters used for model fitting across all responses. The *R*^2^ values for the polynomial model, representing the inactivation of *S. cerevisiae* (*Y*_1_), PPO inactivation (*Y*_2_), and retention of vitamin C (*Y*_3_), were 0.93, 0.97, and 0.97, respectively, with corresponding adjusted *R*^2^ values of 0.91, 0.96, and 0.96. These values indicate a strong fit of the data to the model for each response. Additionally, the model *p*-values for all responses were significantly lower than 0.0001, accompanied by higher *F*-values (51.9, 133.6, and 122.4 for *Y*_1_, *Y*_2_, and *Y*_3_, respectively). An insignificant lack of fit (*p*-value > 0.15) suggests that the variations in responses are primarily influenced by process variables rather than random noise. In conclusion, the generated equation demonstrates a robust fit and reliability, facilitating the understanding of the relative impact of process variables (linear terms) and various combined effects between variables (quadratic and interaction terms) during the juice processing.

##### Linear Terms

The linear terms (*x*_1_, *x*_2_, and *x*_3_) within the quadratic model have demonstrated significance (*p* < 0.05), indicating their notable contribution to the observed variations in the three responses, as illustrated in Table 4. It is worth noting, however, that the linear term associated with pH did not exhibit significance (*p* > 0.1) in the context of *S. cerevisiae* inactivation. The effective fluence of PL exposure is found to have the largest effect on *S. cerevisiae* and PPO inactivation when the coefficients for these linear terms are examined. Moreso than PL effective fluence, ultrasound intensity affects vitamin C retention. With the exception of pH, PL effective fluence and US intensity both favorably influence *S. cerevisiae* and PPO inactivation. A positive influence in this case means that a higher response value results by an increase in the independent variable’s magnitude. On the other hand, *S. cerevisiae* inactivation, PPO, and vitamin C retention are adversely affected by the pH of the matrix. This suggests that increasing the juice’s pH might decrease vitamin C retention and minimize the inactivation of *S. cerevisiae* and PPO. The three linear terms (*x*_1_, *x*_2_, and *x*_3_) all have a negative effect on the retention of vitamin C. Gomez-Lopez et al. [7] found that UV-induced DNA damage causes PL-induced microbial inactivation, while sonic waves break cell membranes in US inactivation. Changing inactivation methods may increase their levels. PPO accelerates the oxidation of the polyphenols in the juice, contributing to the nutritional and sensorial quality [29]. Both the duration and intensity of the ultrasound treatment had significant effects on enzyme inactivation. Enzymes may lose function if US waves alter their secondary and tertiary structures. Sonochemical processes during US treatment generate free radicals that may accelerate ascorbic acid oxidation [30].

##### Square Terms

Several square terms present in the polynomial models contribute to providing insight into the overall relationship between process variables and responses. In the case of the inactivation of *S. cerevisiae*, only the square term of PL effective fluence (coefficient = 0.053) is significant (Table 4). For PPO inactivation, all three square terms contribute; moreover, the square term for PL fluence is 1.71. However, the coefficients for pH and US intensities are 1.32 and 1.15, respectively. The only square term significant for vitamin C retention is US intensity, with a coefficient of −0.17. When the variable increases up to a maximum, a negative square term indicates that the response value increases and then trends in the opposite direction. Conversely, a positive square term indicates that an increase in the variable will initially compromise the response value; however, after the behavior reaches the optima, the opposite behavior becomes dominant.

##### Interaction Terms

**Figure 2:**
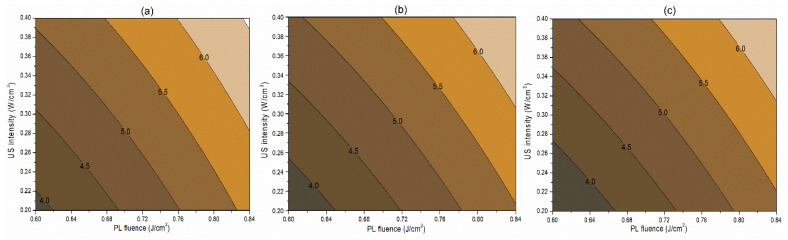
Contour plots showing the influence of pulsed light (PL) and ultrasound (US) conditions on the inactivation of *S. cerevisiae* (log cfu/mL) in the juice. (**a**) Matrix pH 3.5, (**b**) matrix pH 4.0, and (**c**) matrix pH 4.5.

**Figure 3:**
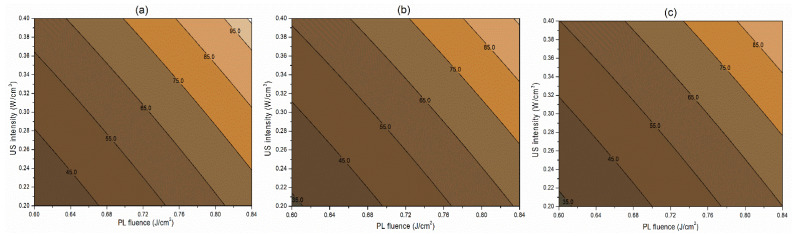
Contour plots showing the influence of pulsed light (PL) and ultrasound (US) conditions on the inactivation of PPO (%) in the juice. (**a**) Matrix pH 3.5, (**b**) matrix pH 4.0, and (**c**) matrix pH 4.5.

**Figure 4:**
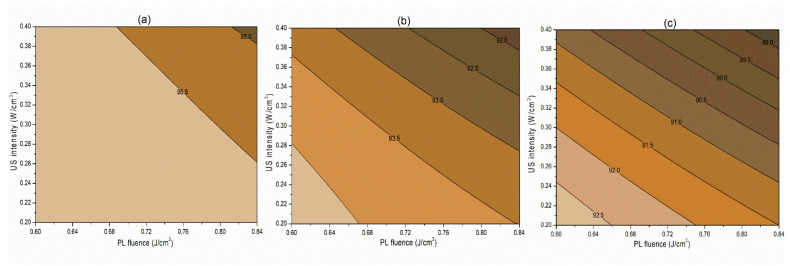
Contour plots showing the influence of pulsed-light and ultrasound conditions on the retention of vitamin C (%) in the juice. (**a**) Matrix pH 3.5, (**b**) matrix pH 4.0, and (**c**) matrix pH 4.5.

The interaction term (−0.06) between PL effective fluence (*x*_2_) and US intensity (*x*_3_) influencing the inactivation of *S. cerevisiae* is significant at *p* < 0.1. A negative interaction coefficient reflects that PL effective fluence and US intensity are acting antagonistically to a minimal extent (Table 4). There may be chances of US intensity protecting the inactivation of *S. cerevisiae*. The slight concave nature of the *Y*_1_ contours between PL fluence and US intensity at different pH levels reflects the same in Figure 2. On the other hand, the PL effective fluence and US intensity behave synergistically (coefficient 1.97) for PPO inactivation (Figure 3). This means an increase in the PL effective fluence and US intensities leads to a higher PPO inactivation. For vitamin C retention, the interactions of pH-PL, pH-US, and PL-US showed an antagonistic (negative coefficients) trend toward the response. An increase in pH-PL, pH-US, and PL-US would lead to greater vitamin C loss (Figure 4). Ferrario et al. [31] observed that 60 s PL at 71.4 J/cm^2^ followed by 30 min US reduced *S. cerevisiae* by 6.4 and 5.8 log cycles in commercial and natural apple juice, respectively. Combining US and PL with natural apple juice reduced *E. coli* survival by 5.4 logs. In another case, US (600 W, 20 kHz, 30 min, and beginning temperature: 44 °C) and PL (0.73 J/cm^2^, 155 mL/min, and temperature build-up from 44 to 56 °C) reduced *S. cerevisiae* cells by 6.4 and 5.8 log cycles in commercial and natural apple juice, respectively [32]. This demonstrated heat-induced inactivation. Wang et al. [29] found US-UV at 10 min, 600 W inactivated mango juice PPO, POD, and PME. Enzyme inactivation is caused by structural changes to enzyme proteins, typically in the tertiary structure. Unfolding, aggregation, protein backbone cleavage, or the all-or-nothing approach to these processes may cause these changes [33]. Fonteles et al. [34] found that ultrasound and ozone synergistically improved cashew apple juice independent of order. Parameters such as oxygen, pressure, temperature, metal ions, and pH contribute to vitamin C degradation [35].

#### 3.2.2. Numerical Optimization

In the pursuit of ensuring the safety, stability, and quality of the juice, our objective was to optimize the inactivation of *S. cerevisiae*, PPO, and vitamin C retention through numerical optimization. To prioritize these objectives, the relative importance (*r_i_*) was assigned on a scale of 1 to 5, with 5 signifying the highest importance. Vitamin C retention and PPO inactivation received a significant priority rating of 4 out of 5, underscoring their importance. Meanwhile, *S. cerevisiae* inactivation was accorded the utmost significance, with the highest relative importance (*r_i_*) score of 5, reflecting our primary concern for microbial safety (Table 5). The numerical optimization suggested that sweet lime juice with pH 3.5 treated at PL effective fluence of 0.80 J/cm^2^ followed by US intensity of 0.4 W/cm^3^ would achieve 6.2 log cycles inactivation of *S. cerevisiae*, 90.1% PPO inactivation, and 95.0% retention of vitamin C, with an overall desirability of 0.89. The validation experiment was conducted at juice pH 3.5/0.80 J·cm^−2^/0.4 W·cm^−3^. The observed value of *S. cerevisiae* inactivation was 6.1 ± 0.2 log cfu/mL with a 90.5% inactivation of PPO and 95% retention of vitamin C (Table 6). The predicted and actual values are almost similar, thus validating the numerical optimization. 

### 3.3. Quality Attributes of the Optimized PL + US Treated Sweet Lime Juice

The characteristics that define the quality of the chosen juice samples, including untreated juice (S1), optimally pulsed-light + ultrasound-treated juice (0.80 J/cm^−2^ + 0.4 W cm^−3^) (S2), PL-treated juice (effective fluence of 1.2 J/cm^2^) (S3) [14], US-treated juice (0.7 W/cm^3^) (S4) [15], and thermally treated juice (95 °C/5 min) (S5) are summarized in Table 6.

#### 3.3.1. Microbial Inactivation

Compared to the untreated juice (S1), the complete inactivation of aerobic mesophiles, yeasts and molds, *E. coli*, *L. monocytogenes,* and *S. cerevisiae* was achieved in the S2, S3, and S4 samples. After thermal pasteurization (95 °C/5 min), the juice (S5) was microbially safe, with no AM, YM, *E. coli*, *L. monocytogenes*, or *S. cerevisiae* detected (Table 6). Several authors have reported similar findings. For example, Alabdali et al. [36] discovered that a 50 °C, 3.5 L/min flow rate, 5.1 mW/cm^2^ UV dosage, and 10 min US (200 W) reduced the microbial population to below detection limits. There is evidence of applying sequential nonthermal hurdles to achieve microbial inactivation in food matrices. For instance, Ferrario et al. [37] discovered that 60 s of PL at 71.4 J/cm^2^, followed by 30 min of US, reduced *S. cerevisiae* by 6.4 and 5.8 log cycles, respectively, in commercial and natural apple juice. According to Gomez-Lopez et al. [7], UV-induced DNA damage (photochemical effect) is the primary cause of PL-induced microbial inactivation. In contrast, US inactivation is caused by sonic waves physically destroying cell membranes.

#### 3.3.2. Enzyme Inactivation

The percentages of PPO inactivation were 90.5%, 99.9%, 60%, and 99% in S2, S3, S4, and S5, respectively. The values pertaining to the inactivation of peroxidase (POD) were 95.3%, 99.9%, 95.5%, and 100% in samples S2, S3, S4, and S5, respectively. Similarly, the percentages for PME inactivation were 97.6%, 99.9%, 99.8%, and 100% in S2, S3, S4, and S5, correspondingly (Table 6). Spoilage enzyme activity (PPO, POD, and PME) in the pasteurized juice was less than 0.5%, ensuring enzymatic stability. Similar pasteurization conditions have been recommended in prior studies for achieving microbial and enzymatic stability in fruit beverages. According to Iqbal et al. [38], structural changes to enzyme proteins, particularly in the tertiary conformation, cause enzyme inactivation. These alterations are probably a result of processes like unfolding, aggregation, cleavage of the protein backbone, or a sudden transition to complete unfolding and aggregation.

#### 3.3.3. Physicochemical Attributes

There is no significant change in pH, TSS, and TA after S2, S3, S4, and S5, respectively. Regarding physicochemical properties, Ferrario et al. [9] discovered similar trends in apple juice. The sequential PL and US intensities were insufficient to cause the pH and TA to change significantly. PL + US energy levels and processing time were most likely insufficient to disrupt chemical bonds between dietary components. TSS may not have changed significantly after PL and US treatments [39]. 

#### 3.3.4. Bioactive Compounds

The total phenolic content decreased by 4.1% and 6.8% in the S2 and S3 samples, respectively. In comparison, a 16.6% increase in phenolic content and a 9.6% increase in antioxidant capacity were reported in the S4 sample. The antioxidant capacity decreased by 3.9% and 8.35 in the S2 and S3 samples, respectively. The vitamin C content decreased by 4.9% and 22.3% in the S2 and S3 samples, respectively. In comparison, an increase of 14.8% was observed in the S4 sample. Thermal pasteurization did not significantly impact the TSS but resulted in a 38.6% reduction in total phenolic content, a 42.2% loss in antioxidant capacity, and a 42.3% degradation in vitamin C content in the sample (S5). In previous studies, ultraviolet light and ultrasound were reported to preserve the antioxidants in mango juice [30]. Another explanation for the antioxidant capacity of juice is PL exposure and cavitation-produced hydroxyl radicals, which may add a second hydroxyl group to a phenolic molecule’s benzoic ring [40]. Fonteles et al. [34] discovered that applying ultrasound and ozone in any order had a synergistic effect on microbial inactivation of cashew apple juice. The degradation of vitamin C can be influenced by various factors, such as oxygen, elevated temperature, pressure, metal ions, and pH. Additionally, the UV-C wavelength emitted by PL induces photo-oxidation, thereby diminishing the quality of juice treated with PL [35]. To conclude, sequential PL and US processing could be a better alternative, considering the safety, stability, and nutrient retention in sweet lime juice. 

#### 3.3.5. Color Profile and Bioactive Compounds

The untreated juice sample showed the color values of L* = 65.02 ± 0.03, a* = − 4.12 ± 0.05, and b* = 10.93 ± 0.06, while the sequentially treated sample (S2) had color values of L* = 63.61 ± 0.02, a* = − 4.95 ± 0.03, and b* = 10.64 ± 0.04. The browning index values were 63.8, 64.7, 67.1, 64.0, and 72.6 in S1, S2, S3, S4, and S5, respectively. The total color change values were 1.7, 7.8, 2.6, and 12.4 for S2, S3, S4, and S5, respectively. The thermally pasteurized juice showed a darker color (increased ΔE* and reduced L* value) and greater redness (increased a* value), possibly due to phenolic isomerization or decomposition. The alteration in color was clearly discernible, signifying the adverse influence of intense thermal processing on color integrity. Additionally, thermal pasteurization led to the loss of vitamin C and total phenolics in various citrus juices, as reported in previous studies. Compared to thermally pasteurized juice, PL-, US-, and PL + US-pasteurized juices retained more phenolics, antioxidants, and vitamin C, in line with earlier research [13,14]. The thermal pasteurization procedure could potentially trigger thermal degradation, acid-catalyzed degradation, and/or the Maillard reaction, resulting in a decline in the phytochemical content in the citrus juice. Caminiti et al. [41] discovered a 4.51 overall color shift in orange and carrot juice following PL and manothermosonication. The color of sweet lime juice may change due to ultrasound cavitation and photo-oxidation. These processes can result in physical, chemical, and biological phenomena like increased diffusivities, particle breakdown, and carotenoid conformational change, which results in colorless pigments [42].

#### 3.3.6. Viscosity

The viscosity of fresh sweet lime juice was 12.25 ± 0.3 cP, which is low compared to other fruit beverages, such as mango juice, banana juice, papaya juice, sapota juice, etc. The viscosities of untreated, sequential PL + US, and thermally treated sweet lime juice are 12.25 ± 0.3, 12.05 ± 0.2, and 12.17 ± 0.3 cP, respectively. The viscosity of sweet lime juice did not change with respect to sequentially pulsed light + ultrasound and thermal treatments. Sweet lime juice’s viscosity is almost near the viscosity of distilled water (~10.00 cP) [43]. 

#### 3.3.7. Overall Sensory Acceptability

The sensorial overall acceptability values are 8.1, 7.7, 7.2, 7.5, and 6.9 out of 9 in S1, S2, S3, S4, and S5, respectively. To be more specific, the flavor, mouthfeel, and aroma of the optimized PL + US sample were more comparable to those of untreated sample. For instance, the flavor, mouthfeel, and aroma of the optimized PL + US sample were 7.5 ± 0.2, 7.4 ± 0.3, and 7.5 ± 0.2, while the corresponding values for the untreated sample were 7.8 ± 0.1, 7.9 ± 0.2, and 8.2 ± 0.1 respectively. In contrast, when compared to the traditional thermal pasteurization method, the citrus juice subjected to sequential PL + US treatment received a higher hedonic rating. The reduced overall acceptability of the thermally treated sample can be attributed to browning, which arises from the copolymerization of organic acids. Anjaly et al. [44] stated the combination of ultrasound treatment (33 kHz) for 22.95 min and ultraviolet dosage of 1.577 J/cm^2^ was found to retain the organoleptic quality close to that of fresh pineapple juice. 

### 3.4. Characterization of Optimized PL + US Treated Sweet Lime Juice

#### 3.4.1. Phenolic Profiling of Sweet Lime Juice

HRLC-MS of the optimally treated juice identified 15 phenolic compounds (Table 7) in sweet lime juice by comparing mass spectrometry data with characteristic fragment ions. Phenolic compounds, such as 3beta, 6beta-dihydroxynortropane (RT: 1.107), dihydrocaffeic acid 3-O-glucuronide (RT: 1.341), naringenin (RT: 4.978), isocitrate (RT: 1.483), dalpanin (RT: 8.973), and kuwanon Z (RT: 4.701), were identified in both the untreated and PL + US-treated samples (0.8 J/cm^2^ + 0.4 W/cm^3^). Vinyl caffeate (RT: 4.616) and 2-(2,5-dimethoxyphenyl)-5,6,7,8-tetramethoxy-4H-1-benzopyran-4-one (RT: 10.235) were the other compounds that were detected in only the treated sample. PL + US treatment degraded 1,2-dihydrostilbene (RT: 3.284), isomyristicin (RT: 4.641), biorobin (RT: 4.717), and 7b-hydroxy-3-oxo-5b-cholanoic acid (RT: 19.317). PL + US forms the following compounds: 3beta,6beta-dihydroxynortropane, a tropane alkaloid; dihydrocaffeic acid 3-O-glucuronide, a powerful antioxidant; naringenin, a flavonoid with strong anti-inflammatory and antioxidant properties; isocitrate, an isomer of citric acid; dalpanin, a flavonoid; kuwanon Z, a flavan; and vinyl caffeate, an antioxidant. 2-(2,5-dimethoxyphenyl)-5,6,7,8-tetramethoxy-4H-1-benzopyran-4-one is a methoxyflavone with methoxy groups at 5, 6, 7, 8, 3′, and 5′ [45]. The enhanced preservation of these phenolic components can be attributed to the transformation of insoluble, bound phenolic compounds into soluble, free phenolic compounds induced by pulsed light and ultrasound. Furthermore, this preservation results from the inactivation of PPO and the removal of obstructive oxygen from the sample during ultrasound treatment [30]. In the course of PL + US processes, the hydroxyl radical may hydroxylate the ortho-, meta-, and para-positions of phenolic aromatic rings, thereby altering the composition of the sample [39].

#### 3.4.2. Influence of PL + US Treatment on the Morphology of *Saccharomyces cerevisiae*

**Figure 5:**
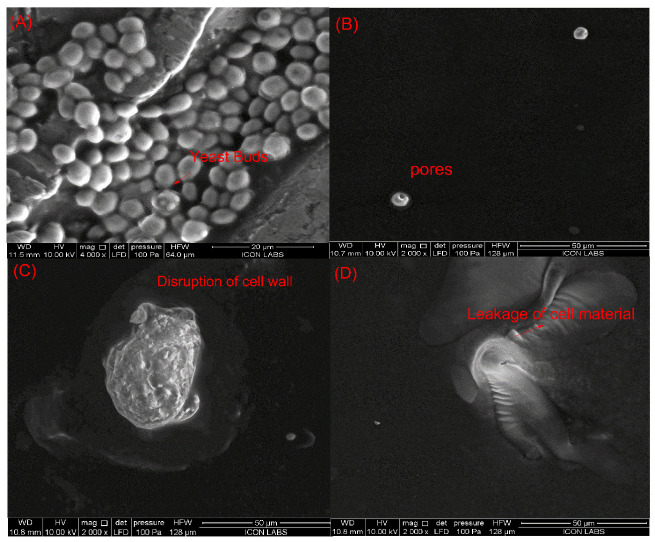
Influence of individual and sequential treatments on the morphology of the *Saccharomyces cerevisiae* (**A**) untreated sample; (**B**) pulsed light sample (0.84 J/cm^2^); (**C**) ultrasound sample (0.4 W/cm^3^); (**D**) PL + US (0.60 J/cm^2^ + 0.4 W/cm^3^).

*Saccharomyces cerevisiae* is the most resistant microorganism in the juice when compared to *E. coli* and *L. monocytogenes*. Therefore, the juice sample treated with sequential pulsed-light (0.60 J/cm^2^ of effective fluence) and ultrasound (intensity of 0.4 W/cm^3^) treatments (condition at which the inactivation of >5 log cfu/mL was achieved) was studied by SEM to explore possible structural damage in yeast cells. Figure 5 shows the morphology of untreated and PL + US-treated *Saccharomyces cerevisiae* ATCC 9763 cells in sweet lime juice. Regarding untreated *S. cerevisiae* cells (Figure 5A), they exhibited an ellipsoidal shape with intact membranes, organelles, and cell walls. Individual PL treatment (0.84 J/cm^2^) (condition at which a >5-log cycle reduction was achieved) showed pores on the cell layers, while individual US treatment (0.4 W/cm^3^) (highest US condition explored in this study) ruptured the cell wall. PL + US (0.8 J/cm^2^ + 0.4 W/cm^3^) caused a fragmented lumen, punctured cell walls, and cytoplasmatic membrane discontinuities (Figure 5D). Cytoplasmic membrane shrinkage may have reduced semi-permeability, upsetting the osmotic equilibrium. Cell shape alterations, membrane distortion, and vacuolization may have resulted from irregular high-intensity pulses. Leaking cytoplasm triggers cell death [46]. Takeshita et al. [47] reported rounded cells, plasma membrane deformation, and vacuole expansion after PL exposure of *S. cerevisiae* IFO2347 cells in model medium (1.421 J/cm^2^ and batch mode). In a PL-treated model solution (5 s, 4.95 J/cm^2^, 12 mL, and batch mode), Krishnamurthy et al. [48] observed *S. aureus* ATCC 25,923 cell wall disintegration and cell content leaking. They also noticed plasmalemma breakdown and shrinkage. After sequential pulsed-light (0.8 J/cm^2^) and ultrasound (0.4 W/cm^3^) treatments, yeast cells showed unusual structural changes, indicating additional targets besides membranes.

#### 3.4.3. Conformational Change in PPO after Sequential PL + US Treatment

**Figure 6:**
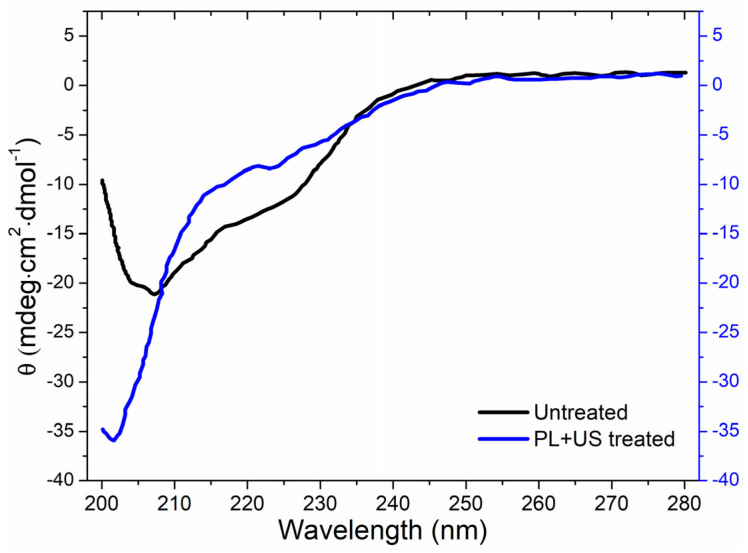
Effect of sequential PL + US (0.8 J/cm^2^ + 0.4 W/cm^3^) on the secondary structure of PPO.

Circular dichroism (CD) analysis revealed the secondary structure (α-helix, β-turn, β-sheet, and random coil) of both untreated and PL + US-treated PPO enzymes (Figure 6). PPO was the most resistant enzyme to the pulsed light domain among all PPO, POD, and PME in the juice. Therefore, as a target enzyme, it is crucial to explore the conformational change in the structure of PPO after sequential PL and US treatment, as well as its individual effect. Sequential pulsed light (0.80 J/cm^2^) and ultrasound (0.4 W/cm^3^) (condition at which a >90% inactivation in the PPO enzyme was achieved) treatments altered the secondary structure. The α-helix structural characteristic absorption peaks at 208 and 222 nm in untreated PPO were negative [49]. Native PPO has a secondary constellation α-helix alignment. Liu et al. [50] found mushroom PPO activity centers had four α-helices crucial to enzyme activity. PL + US processing decreased α-helix content and increased disordered structure, increasing CD spectra negative ellipticity. After PL + US treatment, CD spectra showed an increase in PPO β-sheet concentration at 214 nm. Table 8 compares untreated and PL + US-treated PPO secondary structures. The untreated PPO sample consisted of a 7.7 ± 0.2% α-helix, 37.7 ± 0.4% β-sheet, 6.9 ± 0.2% β-turn, and 47.7 ± 0.5% random coil. Following PL + US treatment, the PPO composition changed to a 2.7 ± 0.1% α-helix, 33.9 ± 0.3% β-sheet, 1.4 ± 0.2% β-turn, and 62 ± 0.7% random coil. PPO’s most critical structural constituent is the α-helix [29]. PL + US treatment inactivates the PPO enzyme, reducing helixes. PL + US increased the random coil ratio in the range of 47.7–62% in PPO. Zhou et al. [51] found a 38.3% α-helix, 12.7% β-sheet, 25.1% β-turn, and 23.9% random coil in a characteristic mushroom PPO. Yi et al. [52] found that the mushroom PPO’s catalytic activity depended on α-helix concentration. These modifications are likely the result of unfolding, aggregation, protein backbone cleavage, or a sudden transition to complete unfolding and aggregation. Enzymes may lose their functionality if US waves alter their secondary and tertiary structures.

## 4. Conclusions

A sequential pulsed light (0.80 J/cm^2^) and ultrasound (0.4 W/cm^3^) treatment can reduce the *S. cerevisiae* population by 6.2 log cycles along with a 90% inactivation of PPO activity and 95% retention of vitamin C in sweet lime juice at pH 3.5. This satisfies the microbial safety aspect (>5-log reduction), enzymatic stability (90% inactivation in resistant enzyme), and nutrient retention (maximal retention of vitamin C) criteria for processing the juice. The total phenolic content and antioxidant activity reduced by 4.1 and 3.9% in the optimized PL + US treated sample, respectively. The viscosity of sweet lime juice did not change with respect to sequentially PL + US and thermal treatments. The sensory acceptability of the optimally PL + US-treated juice (acceptability of 7.7 out of 9) was on par with the untreated one (acceptability of 8.1 out of 9). The optimal PL + US treatment led to fragmented lumens, punctured cell walls, and disruptions in the cytoplasmic membrane of *S. cerevisiae* cells. HRLC-MS confirms that 15 phenolic compounds are retained after PL + US treatment. Inactivation of PPO by PL + US treatment includes losses in α and β helixes and increased disordered structure. Shelf-life stability following PL + US juice processing needs to be explored.

## Figures and Tables

**Table 1:** Dosimetry of the pulsed-light treatments employed for sweet lime juice.

Voltage (kV)	Average Fluence Rate ^#^ (W/cm^2^)	Treatment Time ^§^ (s)	Number of Pulses *	Pulse Width (µs)	Effective Fluence * (F_e_, J/cm^2^) ^¥^
2.8	9.36 ± 0.05	160	160	400	0.60
2.9	10.06 ± 0.02	180	180	400	0.72
2.4	8.74 ± 0.01	240	240	400	0.84
2.7	8.88 ± 0.02	225	225	400	0.80

* The pulse frequency was 1 Hz (1 pulse per second); the ON and OFF times were set at 400 μs. ^#^ Total fluence = average fluence per pulse × number of pulses. ^§^ Fluence rate = total fluence/treatment time. ^¥^ Effective fluence (F_e_, J/cm^2^) = fluence rate × pulse width × number of pulses.

**Table 2:** Effect of PL and US treatment sequence on inactivation of *S. cerevisiae*, PPO, and vitamin C in sweet lime juice at pH 4.0.

Sequence	Treatment Conditions				*S. cerevisiae* Inactivation (log cfu/mL)	PPO Inactivation (%)	Vitamin C Retention (%)
PL + US	0.60	J/cm^2^	0.2	W/cm^3^	3.7 ± 0.1 ^a^	36.3 ± 0.4 ^a^	94.4 ± 0.5 ^a^
	0.84		0.4		6.0 ± 0.2 ^b^	97.2 ± 0.3 ^b^	91.6 ± 0.5 ^b^
US + PL	0.2	W/cm^3^	0.60	J/cm^2^	3.7 ± 0.3 ^a^	36.5 ± 0.4 ^a^	94.2 ± 0.5 ^a^
	0.4		0.84		5.9 ± 0.2 ^b^	97.0 ± 0.5 ^b^	91.4 ± 0.6 ^b^

PL, pulsed light; US, ultrasound; PPO, polyphenol oxidase. The values are presented as mean ± standard error. The different alphabets above each bar denote that the corresponding values differ significantly at a 95% confidence interval of the mean.

**Table 3:** Sequential pulsed-light and ultrasound processing conditions and corresponding responses for the sweet lime juice.

Independent Parameters (Coded Value)			Maximum Temperature Rise (ΔT, °C)		Dependent Variables or Responses (*Y*_1–3_)		
pH	PL Fluence (J/cm^2^)	US Intensity (W/cm^3^)	After PL (J/cm^2^)	After US (W/cm^3^)	Inactivation of *S. cerevisiae* (*Y*_1,_ log cfu/mL)	Inactivation of PPO (*Y*_2,_ %)	Retention in Vitamin C (*Y*_3,_ %)
3.5 (−1)	0.60 (−1)	0.2 (−1)	1.3 ± 0.1	1.0 ± 0.1	3.9 ± 0.3 ^j^	36.8 ± 0.8 ^ef^	96.2 ± 0.4 ^e^
3.5 (−1)	0.60 (−1)	0.3 (0)	2.1 ± 0.2	1.6 ± 0.2	4.6 ± 0.2 ^k^	43.9 ± 1.1 ^g^	95.9 ± 0.6 ^de^
3.5 (−1)	0.60 (−1)	0.4 (+1)	3.5 ± 0.1	2.7 ± 0.1	5.5 ± 0.2 ^l^	50.7 ± 1.0 ^h^	95.5 ± 1.0 ^d^
3.5 (−1)	0.72 (0)	0.2 (−1)	5.6 ± 0.3	4.3 ± 0.1	4.5 ± 0.2 ^k^	47.6 ± 0.9 ^gh^	95.9 ± 0.6 ^de^
3.5 (−1)	0.72 (0)	0.3 (0)	8.9 ± 0.1	6.6 ± 0.2	5.4 ± 0.1 ^ll^	62.5 ± 1.5 ^ij^	95.5 ± 0.8 ^d^
3.5 (−1)	0.72 (0)	0.4 (+1)	10.6 ± 0.1	8.2 ± 0.1	6.0 ± 0.2 ^m^	76.3 ± 1.3 ^j^	95.5 ± 1.2 ^d^
3.5 (−1)	0.84 (+1)	0.2 (−1)	11.4 ± 0.1	8.7 ± 0.2	5.2 ± 0.1 ^kl^	79.9 ± 1.0 ^jk^	95.5 ± 0.7 ^d^
3.5 (−1)	0.84 (+1)	0.3 (0)	13.1 ± 0.1	10.2 ± 0.2	6.0 ± 0.2 ^m^	97.7 ± 0.9 ^kl^	95.5 ± 0.9 ^d^
3.5 (−1)	0.84 (+1)	0.4 (+1)	14.1 ± 0.2	10.9 ± 0.2	6.1 ± 0.1 ^n^	100.0 ± 0.3 ^l^	95.1 ± 1.2 ^d^
4 (0)	0.60 (−1)	0.2 (−1)	1.3 ± 0.1	1.0 ± 0.1	3.7 ± 0.1 ^j^	36.3 ± 0.4 ^ef^	94.4 ± 0.5 ^cd^
4 (0)	0.60 (−1)	0.3 (0)	2.0 ± 0.2	1.6 ± 0.1	4.4 ± 0.1 ^k^	43.6 ± 0.5 ^g^	93.7 ± 0.7 ^cd^
4 (0)	0.60 (−1)	0.4 (+1)	3.8 ± 0.1	2.9 ± 0.1	5.4 ± 0.2 ^l^	50.5 ± 0.6 ^h^	93.0 ± 0.8 ^c^
4 (0)	0.72 (0)	0.2 (−1)	5.7 ± 0.2	4.3 ± 0.1	4.3 ± 0.2 ^jk^	47.3 ± 0.5 ^gh^	94.1 ± 0.9 ^cd^
4 (0)	0.72 (0)	0.3 (0)	8.6 ± 0.1	6.6 ± 0.2	5.2 ± 0.1 ^kl^	62.2 ± 0.4 ^ij^	93.4 ± 1.0 ^cd^
4 (0)	0.72 (0)	0.4 (+1)	10.5 ± 0.2	8.0 ± 0.1	6.0 ± 0.2 ^m^	76.1 ± 0.5 ^j^	92.3 ± 0.9 ^c^
4 (0)	0.84 (+1)	0.2 (−1)	11.5 ± 0.1	8.8 ± 0.1	5.0 ± 0.1 ^jk^	59.9 ± 0.4 ^i^	93.7 ± 0.8 ^cd^
4 (0)	0.84 (+1)	0.3 (0)	13.1 ± 0.1	10.1± 0.2	6.0 ± 0.2 ^m^	79.6 ± 0.5 ^jk^	93.0 ± 0.7 ^c^
4 (0)	0.84 (+1)	0.4 (+1)	14.0 ± 0.2	10.8 ± 0.1	6.0 ± 0.2 ^m^	97.2 ± 0.3 ^kl^	91.6 ± 0.5 ^ab^
4.5 (+1)	0.60 (−1)	0.2 (−1)	1.3 ± 0.4	1.0 ± 0.2	3.6 ± 0.1 ^j^	36.1 ± 0.4 ^f^	92.7 ± 0.6 ^b^
4.5 (+1)	0.60 (−1)	0.3 (0)	2.0 ± 0.3	1.6 ± 0.3	4.3 ± 0.2 ^k^	43.3 ± 0.5 ^g^	91.2 ± 0.7 ^ab^
4.5 (+1)	0.60 (−1)	0.4 (+1)	3.8 ± 0.2	2.9 ± 0.1	5.3 ± 0.2 ^l^	50.2 ± 0.3 ^h^	90.5 ± 0.5 ^ab^
4.5 (+1)	0.72 (0)	0.2 (−1)	5.8 ± 0.2	4.4 ± 0.2	4.2 ± 0.1 ^jk^	47.1 ± 0.4 ^gh^	92.3 ± 0.5 ^b^
4.5 (+1)	0.72 (0)	0.3 (0)	8.4 ± 0.4	6.5 ± 0.3	5.1 ± 0.1 ^kl^	61.8 ± 0.3 ^ij^	90.9 ± 0.6 ^ab^
4.5 (+1)	0.72 (0)	0.4 (+1)	10.4 ± 0.2	8.0 ± 0.2	6.0 ± 0.2 ^m^	75.9 ± 0.7 ^j^	89.8 ± 0.5 ^a^
4.5 (+1)	0.84 (+1)	0.2 (−1)	11.7 ± 0.1	8.7 ± 0.1	4.8 ± 0.2 ^kl^	59.7 ± 0.4 ^i^	92.0 ± 0.5 ^b^
4.5 (+1)	0.84 (+1)	0.3 (0)	13.0 ± 0.2	10.3 ± 0.2	6.0 ± 0.1 ^m^	79.3 ± 0.5 ^jk^	90.2 ± 0.7 ^ab^
4.5 (+1)	0.84 (+1)	0.4 (+1)	13.9 ± 0.2	11.0 ± 0.1	6.0 ± 0.2 ^m^	95.5 ± 0.5 ^k^	89.1 ± 0.4 ^a^
3.5 (−1)	0.60 (−1)	0 (−3)	1.3 ± 0.1	NA	1.9 ± 0.3 ^b^	26.3 ± 0.3 ^de^	95.1 ± 0.5 ^d^
4 (0)	0.60 (−1)	0 (−3)	1.3 ± 0.1	NA	1.6 ± 0.2 ^ab^	25.9 ± 0.4 ^d^	94.0 ± 0.6 ^cd^
4.5 (+1)	0.60 (−1)	0 (−3)	1.3 ± 0.4	NA	1.4 ± 0.2 ^a^	25.8 ± 0.4 ^d^	93.7 ± 0.7 ^cd^
3.5 (−1)	0.72 (0)	0 (−3)	5.6 ± 0.3	NA	3.7 ± 0.2 ^g^	34.0 ± 0.3 ^e^	94.2 ± 0.6 ^cd^
4 (0)	0.72 (0)	0 (−3)	5.7 ± 0.2	NA	3.4 ± 0.2 ^f^	33.8 ± 0.2 ^e^	93.6 ± 0.5 ^c^
4.5 (+1)	0.72 (0)	0 (−3)	5.8 ± 0.2	NA	3.2 ± 0.3 ^ef^	33.6 ± 0.2 ^e^	93.1 ± 0.5 ^c^
3.5 (−1)	0.84 (+1)	0 (−3)	11.4 ± 0.1	NA	5.5 ± 0.2 ^i^	40.2 ± 0.3 ^g^	93.9 ± 0.4 ^cd^
4 (0)	0.84 (+1)	0 (−3)	11.5 ± 0.1	NA	5.3 ± 0.3 ^hi^	38.6 ± 0.2 ^f^	93.4 ± 0.5 ^c^
4.5 (+1)	0.84 (+1)	0 (−3)	11.7 ± 0.1	NA	5.1 ± 0.1 ^h^	42.6 ± 0.3 ^g^	93.0 ± 0.5 ^c^
3.5 (−1)	0 (−6)	0.2 (−1)	NA	1.0 ± 0.1	1.6 ± 0.3 ^ab^	10.5 ± 0.3 ^a^	95.5 ± 0.6 ^d^
4 (0)	0 (−6)	0.2 (−1)	NA	1.0 ± 0.1	1.5 ± 0.3 ^a^	10.4 ± 0.2 ^a^	95.5 ± 0.5 ^d^
4.5 (+1)	0 (−6)	0.2 (−1)	NA	1.1 ± 0.2	1.3 ± 0.2 ^a^	10.3 ± 0.2 ^a^	95.6 ± 0.3 ^d^
3.5 (−1)	0 (−6)	0.3 (0)	NA	6.6 ± 0.2	2.4 ± 0.2 ^d^	13.6 ± 0.2 ^b^	96.7 ± 0.6 ^de^
4 (0)	0 (−6)	0.3 (0)	NA	6.5 ± 0.2	1.8 ± 0.3 ^b^	13.5 ± 0.1 ^b^	96.4 ± 0.4 ^e^
4.5 (+1)	0 (−6)	0.3 (0)	NA	6.7 ± 0.2	1.7 ± 0.2 ^ab^	13.5 ± 0.2 ^b^	95.6 ± 0.3 ^d^
3.5 (−1)	0 (−6)	0.4 (+1)	NA	10.9 ± 0.2	2.9 ± 0.2 ^e^	20.1 ± 0.2 ^c^	97.8 ± 0.7 ^f^
4 (0)	0 (−6)	0.4 (+1)	NA	10.8 ± 0.4	2.5 ± 0.2 ^d^	21.3 ± 0.2 ^c^	97.4 ± 0.4 ^f^
4.5 (+1)	0 (−6)	0.4 (+1)	NA	11.1 ± 0.3	2.0 ± 0.1 ^cd^	17.1 ± 0.1 ^bc^	96.7 ± 0.3 ^de^

PL, pulsed light; US, ultrasound; PPO, polyphenol oxidase; NA, not applicable. Coded values are dimensionless. The values are presented as mean ± standard error. The different alphabets above each bar denote that the corresponding values differ significantly at a 95% confidence interval of the mean. The temperature was measured at the surface of the juice after each individual treatment. The temperature of the juice before treatment was 15 °C.

**Table 4:** Coefficients of polynomial models and corresponding ANOVA data describing the effect of different matrix pH and process parameters on the responses in sweet lime juice during sequential PL and US treatments.

Model Terms	Responses		
	Inactivation of *S. cerevisiae* (*Y*_1_, log cfu/mL)	Inactivation of PPO (*Y*_2_, %)	Retention in Vitamin C (*Y*_3_, %)
	Coefficient ± CI	Coefficient ± CI	Coefficient ± CI
Constant	5.07 ± 0.14	60.13 ± 1.48	93.36 ± 0.12
*x*_1_	−0.11 ± 0.11 **	−2.47 ± 1.09	−2.27 ± 0.09
*x*_2_	0.84 ± 0.10	18.13 ± 1.01	−0.57 ± 0.08
*x*_3_	0.56 ± 0.11	13.11 ± 1.08	−0.67 ± 0.09
*x*_1_ × *x*_2_	0.03 ± 0.04 **	−0.46 ± 0.36 **	−0.30 ± 0.03
*x*_1_ × *x*_3_	0.03 ± 0.06 **	−0.51 ± 0.64 **	−0.54 ± 0.05
*x*_3_ × *x*_2_	−0.06 ± 0.03	1.97 ± 0.33	−0.21 ± 0.02
*x*_1_ × *x*_1_	0.03 ± 0.15 **	1.38 ± 1.53 **	0.04 ± 0.12 **
*x*_2_ × *x*_2_	0.05 ± 0.02	1.71 ± 0.18	−0.01 ± 0.01 **
*x*_3_ × *x*_3_	0.00 ± 0.01 **	1.15 ± 0.44	−0.17 ± 0.03
*p*_lof_	0.155	0.251	0.347
*p*_model_	<0.0001	<0.0001	<0.0001
*F* value	51.9	133.6	122.4
*R^2^*	0.93	0.97	0.97
Adj *R^2^*	0.91	0.96	0.96

PPO, polyphenol oxidase; lof: lack of fit; Adj: adjusted; CI, 95% confidence interval; *x*_1_, *x*_2_, and *x*_3_ are the dimensionless coded forms of pH, pulsed light fluence, and ultrasound intensity, respectively. All terms are significant at *p* < 0.05, otherwise marked as **. ** *p* > 0.10.

**Table 5:** The set of constraints for different parameters targeting high-quality sweet lime juice obtained through sequential pulsed-light and ultrasound processing.

Parameters	Goal	Lower Limit (*L_i_*)	Upper Limit (*U_i_*)	Importance (*r_i_*)	Optimized Value at *D* = 0.89	Actual Value
pH (-)	In range	3.5	4.5	-	3.5	3.5
PL fluence (J/cm^2^)	In range	0.60	0.84	-	0.80	0.80
US intensity (W/cm^3^)	In range	0.2	0.4	-	0.4	0.4
Inactivation of *S. cerevisiae* (*Y*_1_, log cfu/mL)	Maximize	1.3	6.2	5	6.2	6.1 ± 0.2
Inactivation of PPO (*Y*_2_, %)	Maximize	10.3	100	4	90.1	90.5 ± 1.3
Retention in vitamin C (*Y*_3_, %)	Maximize	89.1	97.8	4	95.2	95.0 ± 0.8

PPO, polyphenol oxidase; PL, pulsed light; US, ultrasound; D, overall desirability.

**Table 6:** Changes in biochemical attributes of microbially safe and enzymatically stable sweet lime juice (pH 3.5) during optimized PL and US treatment conditions.

Attributes	Sample Treated at Various Conditions				
	Untreated	PL (*F*_e_ = 0.8 J·cm^−2^) + US (0.4 W·cm^−3^)	PL (*F*_e_ = 1.2 J·cm^−2^) [14]	US (0.69 W·cm^−3^) [15]	Thermal Treatment (95 °C/5 min)
	S1	S2	S3	S4	S5
Aerobic mesophilic count (log cfu/mL)	6.0 ± 0.2	<DL	<DL	<DL	<DL
Yeast and molds count (log cfu/mL)	6.3 ± 0.2	<DL	<DL	<DL	<DL
*E. coli* population (log cfu/mL)	7.0 ± 0.1	<DL	<DL	<DL	<DL
*L. monocytogenes* population (log cfu/mL)	7.0 ± 0.3	<DL	<DL	<DL	<DL
*S. cerevisiae* population (log cfu/mL)	7.1 ± 0.2	<DL	<DL	<DL	<DL
Inactivation of PPO (%)	0 ^d^	90.5 ± 1.1 ^b^	99.9 ± 0.2 ^c^	60.0 ± 1.2 ^a^	99.0 ± 0.3 ^c^
Inactivation of POD (%)	0 ^c^	95.3 ± 1.3 ^a^	99.9 ± 0.1 ^b^	95.5 ± 0.9 ^a^	100 ± 0.1 ^b^
Inactivation of PME (%)	0 ^c^	97.6 ± 0.6 ^a^	99.9 ± 0.1 ^b^	99.8 ± 0.1 ^b^	100 ± 0.1 ^cd^
pH (-)	3.5 ± 0.1 ^a^	3.5 ± 0.1 ^a^	3.5 ± 0.2 ^a^	3.51 ± 0.1 ^a^	3.49 ± 0.2 ^a^
TSS (°Brix)	11.7 ± 0.1 ^a^	11.8 ± 0.2 ^a^	11.7 ± 0.1 ^a^	11.9 ± 0.3 ^a^	11.78 ± 0.3 ^a^
Titratable acidity (% citric acid)	2.1 ± 0.1 ^a^	2.1 ± 0.2 ^a^	2.1 ± 0.1 ^a^	2.1 ± 0.1 ^a^	2.1 ± 0.2 ^a^
Viscosity (cp)	12.25 ± 0.3 ^a^	12.05 ± 0.2 ^a^	N.D.	N.D.	12.17 ± 0.3 ^a^
Total phenolic content (g GAE/L)	26.4 ± 0.3 ^c^	25.3 ± 0.2 ^ab^	24.6 ± 0.2 ^a^	30.8 ± 0.3 ^d^	16.2 ± 0.3 ^a^
Antioxidant capacity (g GAEAC/L)	22.7 ± 0.3 ^c^	21.8 ± 0.3 ^b^	20.8 ± 0.2 ^a^	24.9 ± 0.3 ^d^	13.1 ± 0.2 ^a^
Ascorbic acid (g/L)	2.82 ± 0.2 ^c^	2.68 ± 0.4 ^b^	2.19 ± 0.2 ^a^	3.24 ± 0.3 ^d^	1.64 ± 0.3 ^a^
Browning Index (*BI*)	63.8 ± 0.2 ^a^	64.7 ± 0.3 ^b^	67.1 ± 0.1 ^c^	64.0 ± 0.1 ^a^	72.6 ± 0.4 ^d^
Total color change *(*ΔE*)	-	1.7 ± 0.3 ^a^	7.8 ± 0.4 ^c^	2.6 ± 0.2 ^b^	12.4 ± 0.4 ^d^
Overall acceptability (out of 9)	8.1 ± 0.2 ^c^	7.7 ± 0.2 ^b^	7.2 ± 0.3 ^a^	7.5 ± 0.2 ^ab^	6.9 ± 0.2 ^a^
Flavor (out of 9)	7.8 ± 0.1 ^d^	7.5 ± 0.2 ^c^	6.9 ± 0.1 ^b^	7.1 ± 0.1 ^bc^	6.3 ± 0.3 ^a^
Mouthfeel (out of 9)	7.9 ± 0.2 ^d^	7.4 ± 0.3 ^c^	7.0 ± 0.2 ^b^	7.3 ± 0.2 ^c^	6.1 ± 0.2 ^a^
Aroma (out of 9)	8.2 ± 0.1 ^d^	7.5 ± 0.2 ^bc^	7.1 ± 0.3 ^b^	7.2 ± 0.2 ^b^	6.4 ± 0.3 ^a^

The alphabets in small letters (a, b, c, and d) in the superscripts denote that the mean values are statistically different across the columns at *p* < 0.05. Values are presented as mean ± standard error. PL, pulsed light; US, ultrasound; GAE, gallic acid equivalent; GAEAC, gallic acid equivalent antioxidant capacity; DL, detection limit of 1 log cfu/mL; N.D., not determined.

**Table 7:** Flavor compounds identified in the untreated and the optimized PL + US treated (0.8 J/cm^2^ + 0.4 W/cm^3^) sweet lime juice.

No.	Compound Identified	Untreated Juice (S1)	PL + US-Treated Juice (S2)	RT	Mass	Formula	Description
1	3beta,6beta-Dihydroxynortropane	Present	Present	1.107	143.0956	C_7_H_13_NO_2_	Tropane alkaloid
2	Dihydrocaffeic acid 3-O-glucuronide	Present	Present	1.341	358.0912	C_15_H_18_O_10_	Antioxidants
3	1,2-dihydrostilbene	Present	Absent	3.284	182.109	C_14_ H_14_	Provides protection against chronic diseases
4	Isomyristicin	Present	Absent	4.641	192.0799	C_11_H_12_O_3_	Has anti-cholinergic, antibacterial, and hepatoprotective effects
5	Biorobin	Present	Absent	4.717	594.1595	C_27_H_30_O_15_	Flavones and flavonols
6	Naringenin	Present	Present	4.978	272.0696	C_15_H_12_O_5_	Flavonoids and strong anti-inflammatory and antioxidant activities
7	Hesperetin	Present	Present	6.366	302.08	C_16_H_14_O_6_	Antioxidant and anti-inflammatory properties
8	Ononin	Present	Present	6.438	430.1279	C_22_H_22_O_9_	Isoflavone glycoside
9	Vinyl Cafeate	Absent	Present	4.616	206.0591	C_11_H_10_O_4_	Antioxidant
10	2-(2,5-Dimethoxyphenyl)-5,6,7,8-tetramethoxy-4H-1-benzopyran-4-one	Absent	Present	10.235	402.133	C_21_H_22_O_8_	A methoxy flavone that is flavone substituted by methoxy groups at positions 5, 6, 7, 8, 3′, and 5′
11	N-Hexadecanoylpyrrolidine	Absent	Present	18.837	309.3044	C_20_H_39_NO	Byproduct of the Maillard reaction
12	Isocitrate	Present	Present	1.483	192.027	C_6_H_8_O_7_	Commonly used as a marker to detect the authenticity and quality of fruit products, most often in citrus juices
13	Dalpanin	Present	Present	8.973	534.1754	C_26_H_30_O_12_	Flavonoid
14	7b-Hydroxy-3-oxo-5b-cholanoic acid	Present	Absent	19.317	390.2793	C_24_H_38_O_4_	Bile acid
15	Kuwanon Z	Present	Present	4.701	594.1548	C_34_H_26_O_10_	Flavans that feature a C5-isoprenoid substituent at the 3-position. This could make kuwanon-Z a potential biomarker for consuming these foods

PL, pulsed light; US, ultrasound; RT, retention time. PL + US-treated juice is optimally processed at a PL fluence of 0.8 J/cm^2^, followed by a US intensity at 0.4 W/cm^3^.

**Table 8:** Secondary structure contents of untreated and sequential PL + US-treated (0.8 J/cm^2^ + 0.4 W/cm^3^) polyphenol oxidase enzyme.

Sample	α-Helix (%)	ß Sheet (%)	ß Turn (%)	Random Coil (%)
Untreated (S1)	7.7 ± 0.2	37.7 ± 0.4	6.9 ± 0.2	47.7 ± 0.5
PL + US (S2)	2.7 ± 0.1	33.9 ± 0.3	1.4 ± 0.2	62 ± 0.7

PL, pulsed light; US, ultrasound.

## Data Availability

The original contributions presented in the study are included in the article, further inquiries can be directed to the corresponding author.

## References

[1] Kehinde B.A., Nayik G.A., Rafiq S. (2020). Muntingia calabura. Antioxidants in Fruits: Properties and Health Benefits.

[2] Kashtock M.E. (2004). Guidance for Industry: Juice Hazard Analysis Critical Control Point Hazards and Controls Guidance.

[3] Aneja K.R., Dhiman R., Aggarwal N.K., Kumar V., Kaur M. (2014). Microbes associated with freshly prepared juices of citrus and carrots. Int. J. Food Sci..

[4] Aleem S., Ramteke P.W. (2017). Sensory and Nutritional study of locally available fresh and processed Fruit and Vegetable juices in Allahabad City. Pharma Innov..

[5] Khandpur P., Gogate P.R. (2015). Effect of novel ultrasound-based processing on the nutrition quality of different fruit and vegetable juices. Ultrason. Sonochem..

[6] Chacha J.S., Zhang L., Ofoedu C.E., Suleiman R.A., Dotto J.M., Roobab U., Agunbiade A.O., Duguma H.T., Mkojera B.T., Hossaini S.M. (2021). Revisiting nonthermal food processing and preservation methods—Action mechanisms, pros and cons: A technological update (2016–2021). Foods.

[7] Gómez-López V.M., Ragaert P., Debevere J., Devlieghere F. (2007). Pulsed light for food decontamination: A review. Trends Food Sci. Technol..

[8] Illera A.E., Sanz M.T., Benito-Román O., Varona S., Beltrán S., Melgosa R., Solaesa A.G. (2018). Effect of thermosonication batch treatment on enzyme inactivation kinetics and other quality parameters of cloudy apple juice. Innov. Food Sci. Emerg. Technol..

[9] Ferrario M., Alzamora S.M., Guerrero S. (2015). Study of pulsed light inactivation and growth dynamics during storage of *Escherichia coli* ATCC 35218, *Listeria innocua* ATCC 33090, *Salmonella Enteritidis* MA44 and *Saccharomyces cerevisiae* KE162 and native flora in apple, orange and strawberry juices. Int. J. Food Sci. Technol..

[10] Char C.D., Mitilinaki E., Guerrero S.N., Alzamora S.M. (2010). Use of High-Intensity Ultrasound and UV-C Light to Inactivate Some Microorganisms in Fruit Juices. Food Bioprocess Technol..

[11] Pataro G., Muñoz A., Palgan I., Noci F., Ferrari G., Lyng J.G. (2011). Bacterial inactivation in fruit juices using a continuous flow Pulsed Light (PL) system. Food Res. Int..

[12] Pellicer J.A., Gabaldón J.A., Gómez-López V.M. (2021). Effect of pH on pulsed light inactivation of polyphenol oxidase. Enzym. Microb. Technol..

[13] Namala B., Reddy P.Y. (2017). Design, development and fabrication of batch type continuous UV-C light system for food products. J. Pharmacogn. Phytochem..

[14] Shaik L., Chakraborty S. (2022). Effect of pH and total fluence on microbial and enzyme inactivation in sweet lime (*Citrus limetta*) juice during pulsed light treatment. J. Food Process. Preserv..

[15] Shaik L., Chakraborty S. (2023). Ultrasound processing of sweet lime juice: Effect of matrix pH on microbial inactivation, enzyme stability, and bioactive retention. J. Food Process Eng..

[16] Putnik P., Pavlić B., Šojić B., Zavadlav S., Žuntar I., Kao L., Kitonić D., Kovačević D.B. (2020). Innovative hurdle technologies for the preservation of functional fruit juices. Foods.

[17] Ramírez-Corona N., García N.A., Martínez M.J., López-Malo A., Mani-López E. (2024). Effect of combining ultrasound and UVC treatments for processing orange juice and mango nectar on their microbiological, physicochemical, and sensory characteristics. Innov. Food Sci. Emerg. Technol..

[18] Hasani M., Chudyk J., Murray K., Lim L.T., Lubitz D., Warriner K. (2019). Inactivation of Salmonella, Listeria monocytogenes, Aspergillus and Penicillium on lemons using advanced oxidation process optimized through response surface methodology. Innov. Food Sci. Emerg. Technol..

[19] Gómez-López V.M., Bolton J.R. (2016). An Approach to Standardize Methods for Fluence Determination in Bench-Scale Pulsed Light Experiments. Food Bioprocess Technol..

[20] Guerrouj K., Sánchez-Rubio M., Taboada-Rodríguez A., Cava-Roda R.M., Marín-Iniesta F. (2016). Sonication at mild temperatures enhances bioactive compounds and microbiological quality of orange juice. Food Bioprod. Process..

[21] Sahoo P., Chakraborty S. (2023). Influence of Pulsed Light, Ultrasound, and Series Treatments on Quality Attributes, Pectin Methyl Esterase, and Native Flora Inactivation in Sweet Orange Juice (*Citrus sinensis* L. Osbeck). Food Bioprocess Technol..

[22] Dak M., Verma R.C., Jaaffrey S.N.A. (2007). Effect of temperature and concentration on Rheological properties of ‘Kesar’ mango juice. J. Food Eng..

[23] Rodríguez-Rivera M.P., Lugo-Cervantes E., Winterhalter P., Jerz G. (2014). Metabolite profiling of polyphenols in peels of *Citrus limetta* Risso by combination of preparative high-speed countercurrent chromatography and LC-ESI-MS/MS. Food Chem..

[24] Kelebek H., Selli S., Kola O. (2017). Quantitative determination of phenolic compounds using LC-DAD-ESI-MS/MS in cv. Ayvalik olive oils as affected by harvest time. J. Food Meas. Charact..

[25] Kaláb M., Yang A.-F., Chabot D. (2008). Conventional Scanning Electron Microscopy of Bacteria. Infocus Mag..

[26] Dhar R., Chakraborty S. (2024). Effect of continuous microwave processing on enzymes and quality attributes of bael beverage. Food Chem..

[27] Miles A.J., Ramalli S.G., Wallace B.A. (2022). DichroWeb, a website for calculating protein secondary structure from circular dichroism spectroscopic data. Protein Sci..

[28] Andrade M.A., Chacon P., Merelo J.J., Morán F. (1993). Evaluation of secondary structure of proteins from UV circular dichroism spectra using an unsupervised learning neural network. Protein Eng. Des. Sel..

[29] Zhang J., Yu X., Xu B., Yagoub A.E.A., Mustapha A.T., Zhou C. (2021). Effect of intensive pulsed light on the activity, structure, physico-chemical properties and surface topography of polyphenol oxidase from mushroom. Innov. Food Sci. Emerg. Technol..

[30] Wang J., Liu Q., Xie B., Sun Z. (2019). Effect of ultrasound combined with ultraviolet treatment on microbial inactivation and quality properties of mango juice. Ultrason. Sonochem..

[31] Ferrario M., Guerrero S. (2017). Impact of a combined processing technology involving ultrasound and pulsed light on structural and physiological changes of *Saccharomyces cerevisiae* KE 162 in apple juice. Food Microbiol..

[32] Ferrario M., Alzamora S.M., Guerrero S. (2015). Study of the inactivation of spoilage microorganisms in apple juice by pulsed light and ultrasound. Food Microbiol..

[33] Raso J., Barbosa-Cánovas G.V. (2003). Nonthermal Preservation of Foods Using Combined Processing Techniques. Crit. Rev. Food Sci. Nutr..

[34] Fonteles T.V., Barroso M.K.D.A., Alves Filho E.D.G., Fernandes F.A.N., Rodrigues S. (2021). Ultrasound and ozone processing of cashew apple juice: Effects of single and combined processing on the juice quality and microbial stability. Processes.

[35] Vollmer K., Chakraborty S., Bhalerao P.P., Carle R., Frank J., Steingass C.B. (2020). Effect of Pulsed Light Treatment on Natural Microbiota, Enzyme Activity, and Phytochemical Composition of Pineapple (*Ananas comosus* [L.] Merr.) juice. Food Bioprocess Technol..

[36] Alabdali T.A., Icyer N.C., Ucak Ozkaya G., Durak M.Z. (2020). Effect of Stand-Alone and Combined Ultraviolet and Ultrasound Treatments on Physicochemical and Microbial Characteristics of Pomegranate Juice. Appl. Sci..

[37] Ferrario M., Alzamora S.M., Guerrero S. (2013). Inactivation kinetics of some microorganisms in apple, melon, orange and strawberry juices by high-intensity light pulses. J. Food Eng..

[38] Iqbal A., Murtaza A., Hu W., Ahmad I., Ahmed A., Xu X. (2019). Activation and inactivation mechanisms of polyphenol oxidase during thermal and nonthermal methods of food processing. Food Bioprod. Process..

[39] Ordóñez-Santos L.E., Martínez-Girón J., Arias-Jaramillo M.E. (2017). Effect of ultrasound treatment on visual color, vitamin C, total phenols, and carotenoids content in *Cape gooseberry* juice. Food Chem..

[40] Masuzawa N., Ohdaira E., Ide M. (2000). Effects of ultrasonic irradiation on phenolic compounds in wine. Jpn. J. Appl. Phys..

[41] Caminiti I.M., Noci F., Morgan D.J., Cronin D.A., Lyng J.G. (2012). The effect of pulsed electric fields, ultraviolet light or high intensity light pulses in combination with manothermosonication on selected physico-chemical and sensory attributes of an orange and carrot juice blend. Food Bioprod. Process..

[42] Rodriguez-Concepcion M., Daròs J.A. (2022). Transient expression systems to rewire plant carotenoid metabolism. Curr. Opin. Plant Biol..

[43] Momin S.M.I. (2015). Analysis of Viscosity of Orange Fruit Juice to Ensure the Suitability of Processing Applications. Int. J. Pure Appl. Biosci..

[44] Anjaly M.G., Prince M.V., Warrier A.S., Lal A.N., Mahanti N.K., Pandiselvam R., Thirumdas R., Sreeja R., Rusu A.V., Trif M. (2022). Design consideration and modelling studies of ultrasound and ultraviolet combined approach for shelf-life enhancement of pine apple juice. Ultrason. Sonochem..

[45] Vilas-Boas A.A., Magalhães D., Campos D.A., Porretta S., Dellapina G., Poli G., Istanbullu Y., Demir S., San Martín Á.M., García-Gómez P. (2022). Innovative Processing Technologies to Develop a New Segment of Functional Citrus-Based Beverages: Current and Future Trends. Foods.

[46] Niu L., Liu J., Wang X., Wu Z., Xiang Q., Bai Y. (2022). Effect of Combined Treatment with Cinnamon Oil and petit-High Pressure CO_2_ against Saccharomyces cerevisiae. Foods.

[47] Takeshita K., Shibato J., Sameshima T., Fukunaga S., Isobe S., Arihara K., Itoh M. (2003). Damage of yeast cells induced by pulsed light irradiation. Int. J. Food Microbiol..

[48] Krishnamurthy K., Demirci A., Irudayaraj J. (2008). Inactivation of Staphylococcus aureus in milk and milk foam by pulsed UV-light treatment and surface response modeling. Trans. ASABE.

[49] Kuznetsova I.M., Stepanenko O.V., Turoverov K.K., Zhu L., Zhou J.M., Fink A.L., Uversky V.N. (2002). Unraveling multistate unfolding of rabbit muscle creatine kinase. Biochim. Biophys. Acta Protein Struct. Mol. Enzymol..

[50] Liu W., Liu J., Liu C., Zhong Y., Liu W., Wan J. (2009). Activation and conformational changes of mushroom polyphenoloxidase by high pressure microfluidization treatment. Innov. Food Sci. Emerg. Technol..

[51] Zhou L., Liu W., Xiong Z., Zou L., Liu J., Zhong J., Chen J. (2016). Effect of ultrasound combined with malic acid on the activity and conformation of mushroom (*Agaricus bisporus*) polyphenoloxidase. Enzym. Microb. Technol..

[52] Yi J., Yi J., Dong P., Liao X., Hu X., Zhang Y. (2015). Effect of high-hydrostatic-pressure on molecular microstructure of mushroom (*Agaricus bisporus*) polyphenoloxidase. LWT Food Sci. Technol..

